# Soap-Making

**Published:** 1869-11

**Authors:** 


### SOAP-MAKING.

### A NEW MIXTURE USED IN WASHING CLOTHES.

In Berlin, Prussia, the wash-women use a mixture of two ounces of turpentine and a quarter of an ounce of sal-ammoniac well mixed together.

The mixture is put into a bucket of warm water, in which half a pound of soap has been dissolved. Into this mixture the dirty clothes are immersed during the night and the next day washed.

The most dirty cloth is perfectly freed of all dirt, and after two rinsings in fresh water the cloth has not the least smell of turpentine. The cloth does not require so much rubbing, and fine linen is much longer preserved by it.—*American Polytechnic Journal.*

